# The curvilinear responses of biomass accumulation and root morphology to a soil salt-nitrogen environment reflect the phytodesalination capability of the euhalophyte *Suaeda salsa* L.

**DOI:** 10.3389/fpls.2024.1424766

**Published:** 2024-08-06

**Authors:** Yanyan Wang, Tongkai Guo, Changyan Tian, Zhenyong Zhao, Ke Zhang, Wenxuan Mai

**Affiliations:** ^1^ Xinjiang Institute of Ecology and Geography, Chinese Academy of Sciences (CAS), Ürümqi, China; ^2^ College of Resources and Environment, University of Chinese Academy of Sciences, Beijing, China; ^3^ College of Water Resources and Civil Engineering, China Agricultural University, Beijing, China

**Keywords:** euhalophyte, biomass allocation, root morphology, salt stress, phytodesalination

## Abstract

Under the sufficient nitrogen supply, it is of great significance to investigate the law of biomass allocation, root morphological traits, and the salt absorption capacity of euhalophytes to evaluate their biological desalination in saline soil. Although the curvilinear responses of biomass accumulation and root morphology in response to soil salinity have been recognized, these perceptions are still confined to the descriptions of inter-treatment population changes and lack details on biomass allocation in organs at an individual level. In this study, *Suaeda salsa* was grown in root boxes across a range of soil salt levels. The study showed that their growth and development were significantly affected by soil soluble salts. The law of biomass allocation was described as follows: increased soil soluble salts significantly increased the leaf mass ratio and decreased the stem mass ratio, and slightly increased the root mass ratio among treatments. For individuals at each treatment, leaf mass ratio > stem mass ratio > root mass ratio, except in the control treatment at the flower bud and fruit stages. Biomass responses of the control treatment indicated that salt was not rigorously required for *Suaeda salsa* in the presence of an adequate nitrogen supply, as verified by the correlation between biomass, nitrogen, and soil soluble salt. Salt could significantly inhibit the growth of Suaeda salsa (P<0.01), whereas nitrogen could significantly promote its growth (P<0.01). Root morphology in response to soil soluble salts showed that salt acquisition by the root was highest at a salt level of 0.70%, which corresponds to light saline soil. Consequently, we conclude that phytodesalination by *Suaeda salsa* was optimal in the light saline soil, followed by moderate saline soil.

## 1 Introduction

Euhalophytes, a group of halophytes, can accumulate salt when they grow in saline soil, and their optimal soil salinity is approximately 0.50% NaCl (Chapman, 1942; Wang et al., 2021b). When soil salinity is at a suboptimal concentration, euhalophytes show a curvilinear growth response (Flowers and Colmer, 2008; Ma et al., 2019). The curve responses are reflected in the changes in morphological traits and biomass accumulation of vegetative organs (root, stem, and leaf) because the coordinated development of these organs is an important life-history strategy of plants, and the transport of nutrients and the allocation of assimilative substances among the organs are directly or indirectly driven by environmental factors (Jung et al., 2010; Poorter et al., 2012; Pallas et al., 2016). In saline habitats, the stems or leaves of euhalophytes can be reshaped as major organs of salt accumulation due to their structural and functional properties. For example, succulence is the apparent adaptive characteristic in the stems or leaves of euhalophytes to soil salinity (Jennings, 1968), and the degree of succulent is significantly positively correlated with the dry weight of succulent organs (Ma et al., 2019), indicating that the response of leaf biomass allocation to soil salinity may have a similar curve to its growth trajectory. For euhalophytes with leaf succulence, the stems not only serve as a material transport and mechanical support but can accumulate salt. The water potential gradient caused by salt accumulation differences in roots, stems, and leaves can be the main driving force for transporting water in saline habitats. Therefore, we infer that stems should also respond to different soil salt gradients in a curvilinear manner.

In saline habitats, soil desalination is mainly considered to be the quantity of euhalophyte biomass, but biomass formation is either affected by soil salinity or determined by soil nutrient supply; in particular, nitrogen (N) has been proved to be a critical factor restricting plant growth in saline-alkali land (Ma et al., 2020; Wang et al., 2022), as soil salt can limit the processes of N uptake, transformation, assimilation, and reuse of plants (Kant et al., 2010). Generally, N mainly increases the biomass of leaves and stems, yet under N deficiency or stress conditions, it is more likely to increase the below-ground biomass of plants. Therefore, this experiment put an adequate N supply in place to exclude the effect of soil N deficiency on biomass allocation. With euhalophytes, appropriate soil salinity can promote root growth to absorb soil N, whereas excessive salt can inhibit root biomass. Tolerant salt plants can adjust their root architecture, e.g., root lengthening, to acquire N and thus better acclimate to saline habitats (Wang et al., 2021b). In addition, adequate exogenous N supply can increase the salt tolerance of halophytes (Song et al., 2017; Yao et al., 2021) because sufficient N can alleviate or offset the salt ion toxicity on plant growth by intensifying photosynthetic activities (e.g., increasing the chlorophyll content and photosynthetic rate; Wang et al., 2014; Benzarti et al., 2015), improving osmotic regulation (e.g., synthesizing osmotic substances; Zakery-Asl et al., 2014) and limiting Cl^−^ uptake (Song et al., 2009). Therefore, with an adequate N supply, studies of the biomass allocation responses of euhalophytes to saline habitats is of great guiding significance for predicting their growth and biological desalination potential.

To date, there have been many studies on the aboveground parts of euhalophytes (Wang et al., 2023a, 2023c) and their functions on soil improvement (Liu et al., 2024), but there is relatively little information on the belowground root system (Wang et al., 2021b, 2022). The euhalophyte root is the first portal of salt and N acquisition; its biomass responses and phenotypic changes are closely associated with plant growth and development. Some studies have shown that the root biomass of halophytes decreases with upward salt gradients (Wang et al., 2022), and root morphology and architecture are the results of their responses to the spatial heterogeneity of soil nutrients and salts (Alharby et al., 2018; Belghith et al., 2018; Redelstein et al., 2018; Tolley and Mohammadi, 2020). Salt-nitrogen spatial distribution could significantly regulate the root plasticity of euhalophytes. N can promote taproot growth and lateral root spatial extension (Gruber et al., 2013; Sun et al., 2020), and increase the root lengths of euhalophytes on topsoil under light (0.50% NaCl supplement) on moderate (1.00% NaCl supplement) saline soil, while heavy saline soil (>1.00% NaCl supplement) can accelerate fine root growth in the subsoil regardless of N treatment (Wang et al., 2022). Therefore, studies on the responses of root configuration to soil nutrients and salt can not only predict the salt absorption capacity of roots but determine the optimal soil salt content suitable for euhalophyte growth.

The technique of remediating saline-alkali soil based on halophytes is called phytodesalination (Devi et al., 2016; Farzi et al., 2017; Navarro-Torre et al., 2023; Jurado et al., 2024), which has been extensively applied in coastal, semi-arid, and arid regions due to its environmentally friendly nature and cost effectiveness. In these areas, the intercropping of halophytes with conventional crops (Simpson et al., 2018; Barcia-Piedras et al., 2019; Liang and Shi, 2021; Jurado et al., 2024) or continuous cropping systems (Wang et al., 2021a; Liu et al., 2024), as important technical models, have been gradually established to ensure soil health and improve crop productivity, thereby increasing agricultural sustainability and reducing the negative impacts of soil salinity on food security. For instance, the intercropping of *Arthrocaulon macrostachyum* L. with tomato increases tomato yield and decreases soil salinity (Jurado et al., 2024). *Suaeda salsa (S. salsa)* is a euhalophyte that has the leaf succulence trait and not only has a strong adaptability in saline soil but has excellent phytodesalination performance due to its super accumulation of salt (Wang et al., 2023a, 2023b, 2023c). Currently, it has been widely studied and planted as a model plant for saline soil improvement. Although the research objectives (e.g., growth responses, nutrient utilization, salt tolerance mechanism, salt tolerant-gene excavation, and ecological function) and methods (e.g., medium culture in the greenhouse and scale planting in land) are varied (Wang et al., 2023c), there is a lack of systematic studies on the law of biomass allocation under the different soil NaCl concentrations at the organ, individual and population levels. Therefore, this study mainly analyzes the responses of biomass allocation, root morphological traits, N absorption, and utilization of different soil salt gradients under an adequate N supply to better understand (1) the law of biomass allocation, (2) the responses of root phenotypic traits to soil salinity, (3) the relationship between biomass accumulation and soil N utilization under six NaCl levels, and (4) the potential of phytodesalination.

## 2 Materials and methods

### 2.1 Materials

**Figure 1:**
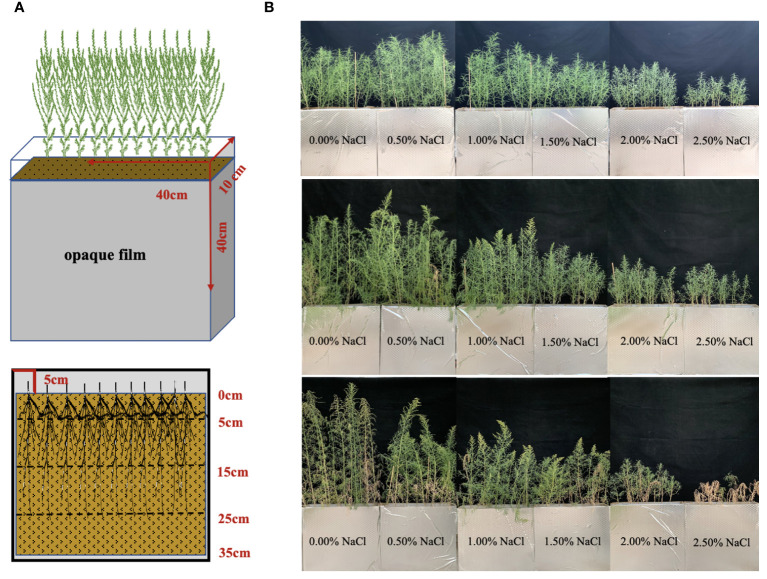
Underground sampling diagram of *S. salsa* **(A)** and apparent character aboveground of *S. salsa* at different growth stages **(B)**.

Seeds of *S. salsa* were collected from the Halophyte Botanical Garden in Karamay (84°59’41.61’’E, 45°28’6.38’’N), Xinjiang Province, China. The topsoil of a cotton field cultivated at Fukang Desert Ecosystem Station (87°45’~88°05’ E, 43°45’~44°30’ N), Chinese Academy of Sciences, was gathered for the root box trial. Initial soil physical and chemical properties were measured, as indicated in **Table 1**. A Root box without drainage made of acrylic plate (40cm ×10cm × 40cm) was used to observe the root system (**Figure 1A**).

**Table 1:** Initial soil physical and chemical properties.

Soil depth	pH	EC	Total soluble salt	Bulk density	Field water capacity	Organic matter	Available N	Olsen- P	Available K
(cm)		(mS cm^-1^)	(g kg^-1^)	(g cm^-3^)	(%)	(g kg^-1^)	(mg kg^-1^)	(mg kg^-1^)	(mg kg^-1^)
0~20	8.26	1.27	2.20	1.32	26.50	11.80	18.54	23.64	560.92

### 2.2 Experimental design

The greenhouse experiment began on June 17, 2022, in Changji, Xinjiang Province, China (87°04’56’’E, 44°09’59’’N). Based on salt percentage as the classification standard of saline-alkali land, six levels of NaCl were used as an experimental treatment design: control treatment (0.20% salt content in the selected soil), 0.70%, 1.20%, 1.70%, 2.20%, and 2.70%, corresponding to 0.50%, 1.00%, 1.50%, 2.00%, and 2.50% NaCl addition accounted for soil weight per root box, respectively. Every treatment was repeated 15 times. Each root box was filled with 18.00 kg of air-dried soil. To supply adequate nutrient for plant growth, fertilizers with 9.54g CO(NH_2_)_2_ and 6.90g KH_2_PO_4_ as basal nutrients were dissolved in 3.80 L of deionized water and applied in the root box. In addition, soil water content was approximately 80% of the field water capacity. After 24 h, 20 intact seeds were sowed evenly in the root box and covered with air-dried soil. When the height of the seedlings was approximately 6 cm, 12 seedlings were kept per root box. Different NaCl levels (0, 90, 180, 270, 360, and 450 g) were dissolved in deionized water and irrigated into the root box every 3 days for a total of five times. During plant growth, the weighing method was applied to replenish deionized water to field water capacity (20%, w/w).

### 2.3 Sample collection and analysis

Plant and soil samples, per five root boxes as a unit, were collected on days 60, 80, and 100, respectively corresponding to the seedling stage, flower bud stage, and fruit stage (**Figure 1B**). The trial was finished on September 26, 2022. After sampling, leaves and stems were parted and put into the oven at 105°C for 30 min and dried at 75°C for 48 h to constant weight. The soil in the root box was divided into four layers (**Figure 1A**): 0–5cm, 5–15cm, 15–25cm, and 25–35cm, respectively. The plant roots were collected in each layer and washed with deionized water. Roots were scanned using a Microtek color platform scanner (Phantom9980XL, resolution 800 dpi). The acquired images were analyzed using FG-RIAS root analysis software to obtain root length (RL) and root average diameter (RAD). Subsequently, the roots were placed in the oven and dried at 65°C to constant weight. Total biomass was calculated as the sum of the roots, stems, and leaves.

Soil samples were air-dried, grinded, and sieved (2 mm and 1 mm). pH and electrical conductivity (EC) were measured using water extracts (pH, 1:2.5 w/v; EC, 1:1 w/v) after suspension for 30 min by a pH meter (S20, Mettler Toledo, Switzerland) and electrical conductivity meter (DDSJ-308A, China). Total soluble salt (TSS) was measured using the residue drying method. Soil available N was measured using an auto analyzer (BRAN LUEBBE AA3, Germany). Phosphorus (Olsen-P) was measured through colorimetric analyses. Available Potassium (K) was measured using an atomic absorption spectrometer (Thermos Electron Corporation, USA). The plant samples (roots, stems, and leaves) were crushed with a ball mill and then digested with H_2_SO_4_-H_2_O_2_ and measured for N concentration using an Automatic Kjeldahl Analyzer (Foss 8400, Denmark). Sodium (Na) was microwave digested with HNO_3_-H_2_O_2_ and measured by inductively coupled plasma atomic emission spectroscopy (735E, Agilent, USA).

### 2.4 Statistical analysis

One-way analysis of variance (ANOVA) followed by the least significant difference (LSD) test were applied to analyze the differences in soil TSS content, biomass, root morphological characteristics (RL and RAD), N and Na concentration, the amount of Na removal of *S. salsa*, and soil residual available N caused by NaCl addition at each growth stage. All statistical analyses were performed using Microsoft Excel and SPSS ver. 29.0 software (IBM Corp. Armonk, NY, USA).

## 3 Results

### 3.1 Biomass

**Figure 2:**
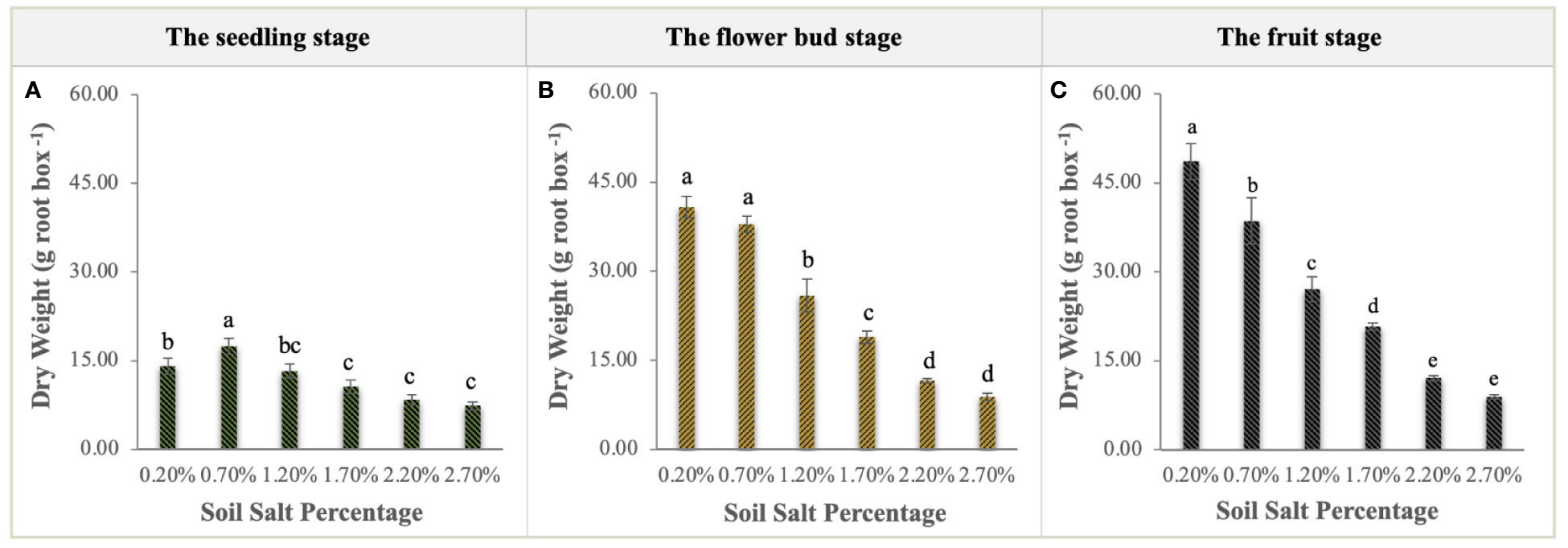
Variations in the biomass accumulation of *S. salsa* at the seedling stage **(A)**, flower bud stage **(B)**, and fruit stage **(C)**. Each treatment had five biological replicates and the assays were repeated five times. Vertical error bars indicate ± SD (N = 5). Different lowercase letters indicate statistically significant differences (P < 0.01).

NaCl had a significant curvilinear effect on the biomass of *S. salsa* (**Figure 2**). At the seedling stage, the total biomass of *S. salsa* growing in the soil with 0.70% salt content was significantly higher than that of the other treatments (P < 0.01), with a maximum of 17.45 g per root box (**Figure 2A**). At the flower bud and fruit stages, the biomass of the control treatment was significantly higher than that of the other treatments (P < 0.01), and the biomass reached 48.65 g per root box at harvest. For the 0.70% salt treatment, biomass decreased slightly at the flower bud stage but significantly at the fruit stage (**Figures 2B, C**). These descriptions of biomass curves indicated that NaCl addition had a significantly negative effect on the growth and development of *S. salsa*.

### 3.2 Biomass allocation in different organs

**Figure 3:**
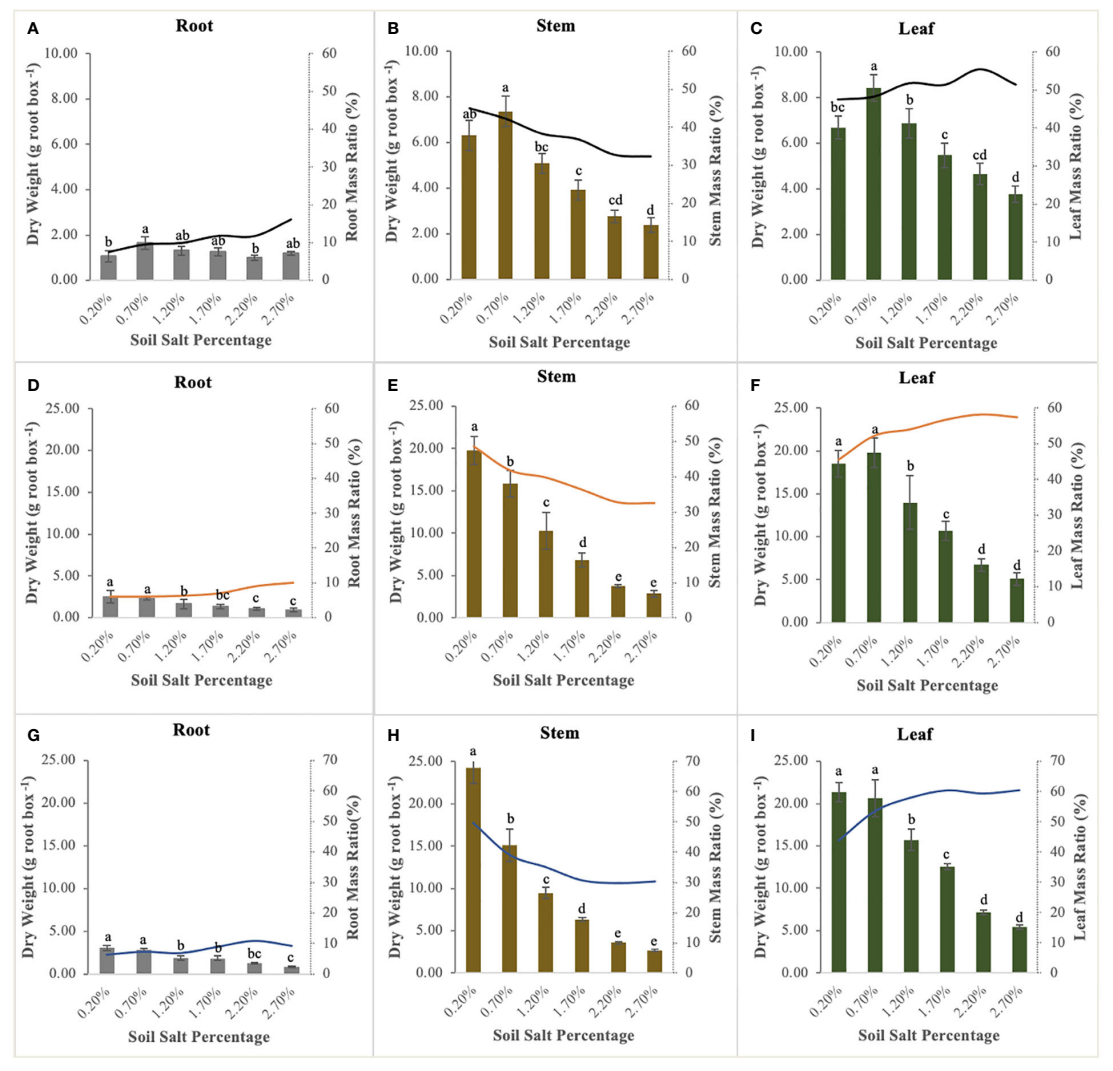
Biomass allocation and mass ratio of root **(A, D, G)**, stem **(B, E, H)**, and leaf **(C, F, I)** at the different sampling stages. Each treatment had five biological replicates and the assays were repeated five times. Vertical error bars indicate ± SD (N = 5). Different lowercase letters indicate statistically significant differences (P < 0.01).

NaCl supplementation significantly affected the biomass accumulation and allocation of the roots, stems, and leaves of *S. salsa*. The biomass accumulation ranking of all organs was as follows: leaf > stem > root (except the control treatment). The stem biomass of the control treatment was higher than the leaf biomass (**Figure 3**), and it was also significantly higher than that of the other treatments; up to 19.79 g and 24.25 g per root box at the flower bud stage and fruit stage, respectively (**Figures 3B, E, H**). Leaf biomass reached the optimum level when NaCl addition accounted for 0.70% of soil weight at the seedling stage and flower bud stage (**Figures 3C, F**). Compared with the significant variations in stem and leaf biomass, root biomass decreased relatively gently, suggesting that root stability is an important survival strategy for *S. salsa* in the salinized soil. In addition, differing from the declining responses of biomass accumulation in all organs to soil soluble salts, the root and leaf mass ratio had an obvious increase (**Figure 3**). The root mass ratio (7.47–16.12%, from the lowest to highest salt levels) at the seedling stage was much higher than that in the other two sampling periods, which were lower, ranging from 6.00–11.00% (**Figures 3A, D, G**). The leaf mass ratio significantly increased with soil salt increment in the fluctuation ranges of 47.6–55.45% at the seedling stage (**Figure 3C**), 45.42–58.20% at the flower bud stage (**Figure 3F**), and 43.85–60.56% at the fruit stage (**Figure 3I**). Conversely, stem mass ratio significantly decreased during the whole growth stage, with the downward ranges from 44.92% to 32.41% at the seedling stage (**Figure 3B**), from 48.53% to 32.50% at the flower bud stage (**Figure 3E**), and from 49.84% to 29.74% at the fruit stage (**Figure 3H**).

### 3.3 Root length and average root diameter

**Figure 4:**
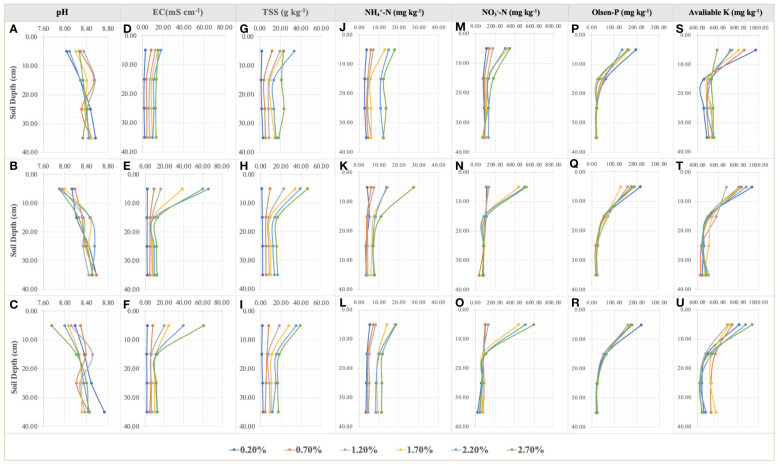
Variations in pH **(A–C)**, EC **(D–F)**, TSS **(G–I)**, NH_4_^+^-N **(J–L)**, NO_3_^–^-N **(M–O)**, Olsen-P **(P–R)**, and available K **(S–U)** in the soil profile at the seedling, flower bud, and fruit stages. Each treatment had five biological replicates and the assays were repeated five times. The scatter values indicate means.

**Figure 5:**
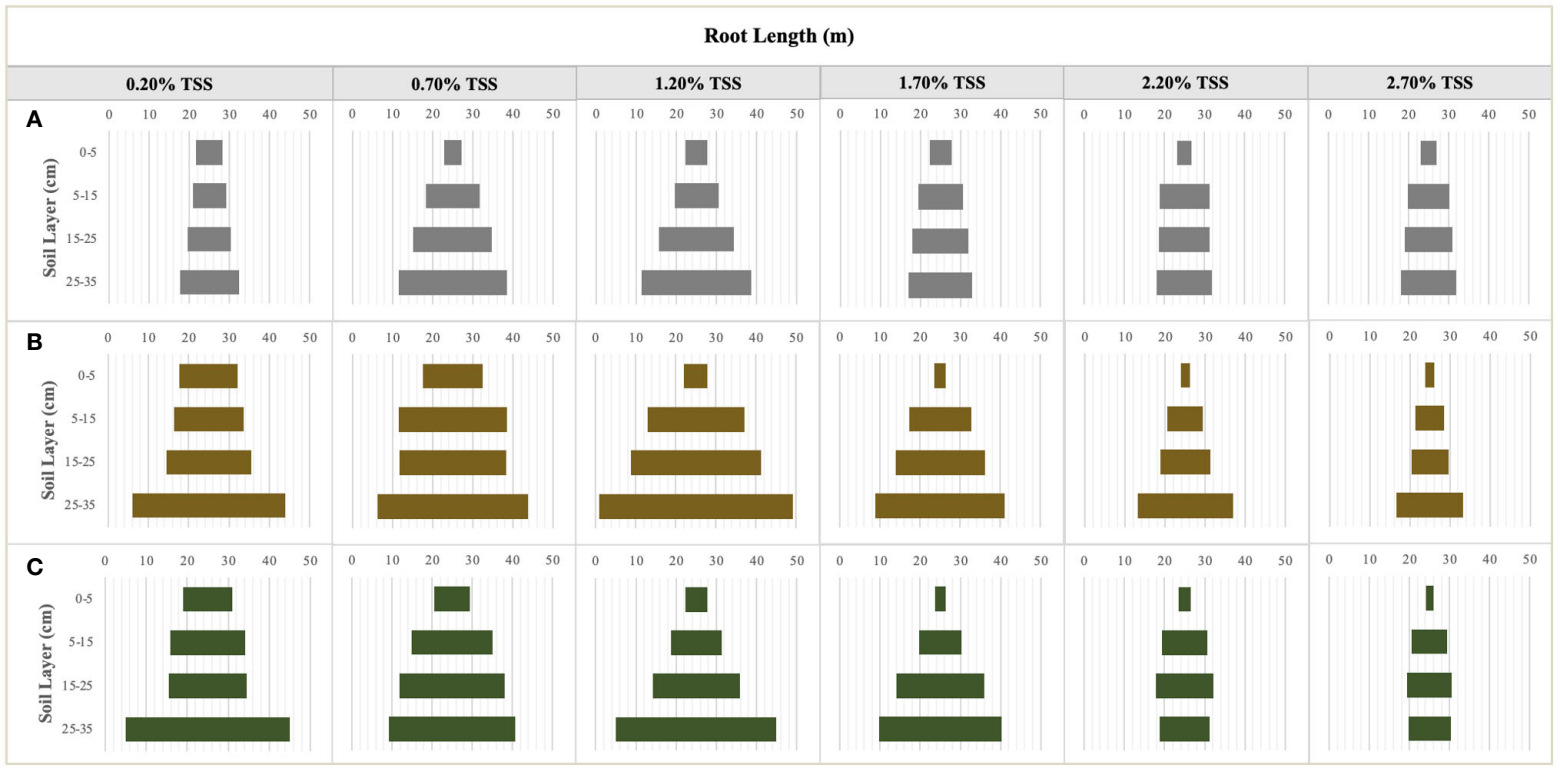
The characteristics of root length distribution in the different soil layers at the seedling **(A)**, flower bud **(B)** an fruit **(C)** stages. Each treatment had five biological replicates and the assays were repeated five times. The horizontal bars indicate the root length means.

NaCl addition provided a heterogenous salinity and nutrient environment for the root growth of *S. salsa* in the different soil layers (**Figure 4**). TSS accumulated significantly on the 0–5cm topsoil, especially in the moderate and high salt treatments (NaCl addition > 1.50%) at the late sampling periods. TSS distribution was comparatively homogeneous in the other soil layers (**Figures 4G–I**). TSS differences in the same soil layers under the different treatments, as important environmental factors significantly led to the changes in root growth and morphology (**Figure 5**). RL layout in the soil matrix was roughly divided into taproots, fine roots, and hairy roots, corresponding to 0−5cm, 5−25cm, and 25−35cm soil layers, respectively (**Figure 5**). When comparing the seedling stage with the flower bud stage (**Figures 5A, B**), soil salt levels of 0.20% and 0.70% increased the RLs of taproots, fine roots, and hairy roots; soil salt levels of 1.20% and 1.70% increased the RLs of fine roots and hairy roots; and soil salt levels of 2.20% and 2.70% increased the RLs of hairy roots but decreased the RLs of taproots and fine roots. At the fruit stage, except for the non-salt treatment, RLs in each soil layer obviously declined, indicating that soil salinity accelerated the aging and death of the root system (**Figure 5C**).

**Figure 6:**
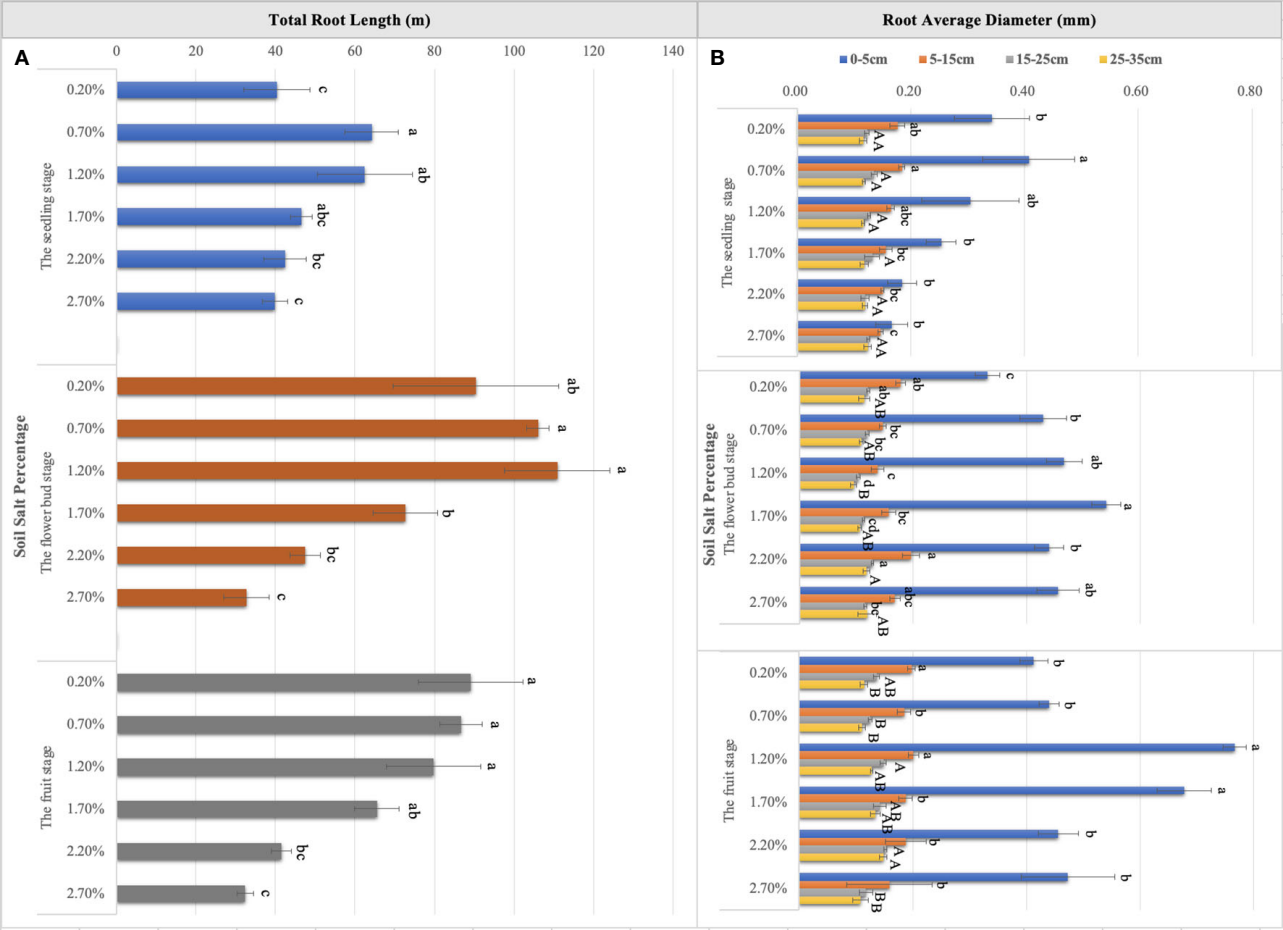
Variations in total root length **(A)** and root average diameter **(B)** in six levels of NaCl treatment. Each treatment had five biological replicates and the assays were repeated five times. The horizontal error bars indicate ± SD (N = 5). Different uppercase and lowercase letters indicate statistically significant differences (P < 0.05 and P < 0.01), respectively.

Throughout the whole growth period, when the supplemental NaCl amount was greater than 1.50%, root growth was markedly inhibited (**Figures 5A–C**), and RL responded to soil TSS in a curvilinear manner. At the seedling stage, total RL reached approximately 64.19 m when soil salt content accounted for 0.70% of the soil. Second, total RL was approximately 62.47 m when soil salt content was 1.20% (**Figure 6A**). At the flower bud stage, a soil salt content of 1.20% induced a RL of up to 110.89 m, and the root was mainly concentrated in the 15–35 cm soil layer. Additionally, the root system grew best during this sampling period (**Figures 5B**, **6A**). At the fruit stage, total RL in the control treatment was higher than that in other treatments, which may be related to the increase of salt to accelerate the aging and death of the root system (P < 0.01; **Figure 6A**). In addition, the change in RAD was mainly reflected in the thickening of taproots in the 0–5 cm soil layer, and RAD also showed an obvious curvilinear response to the addition of exogenous NaCl (**Figure 6B**). At the seedling stage, when the soil salt content was 0.70%, the maximum RAD in the surface soil was approximately 0.41 mm. Although the results at the flower bud and fruit stages showed that the RADs of taproots were higher when soil salt content was greater than 1.20% (**Figure 6B**), they could not represent the actual root thickening because the high salt concentration caused the finer lateral roots to fall off, thereby indirectly increasing their RAD. Root thickening in the other soil stratifications was not obvious with the increasing salt content (**Figure 6B**).

### 3.4 The relationship between root system morphology and soil soluble salt

**Figure 7:**
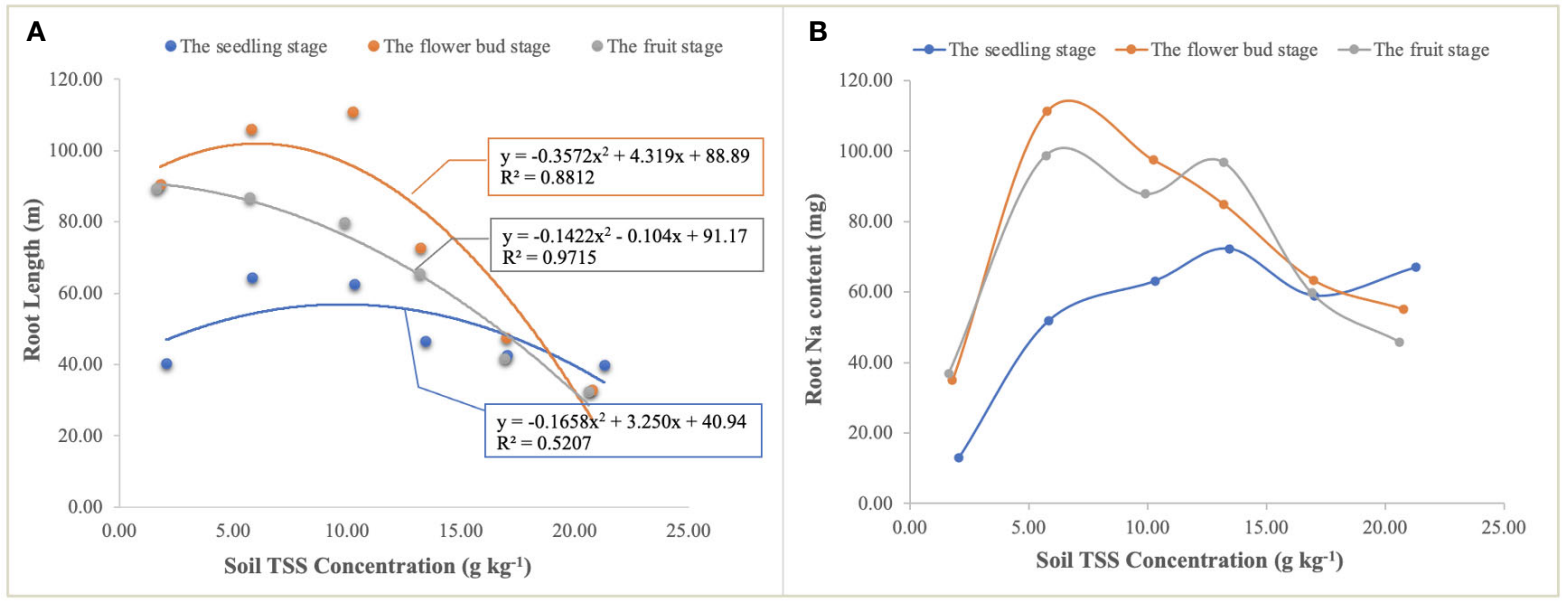
The relationship of quadratic regression between TSS concentration and root length **(A)** and Na uptake by roots under different TSS concentrations in the soil **(B)**. Each treatment had five biological replicates and the assays were repeated five times. TSS concentrations indicate the weighted means of soil salt concentration in four soil layers.

The curve responses of the root to soil TSS were different at the different growth stages (**Figure 7**). At the seedling stage, the correlation of root length in response to soil TSS was significantly lower than that at the flower bud and fruit stages, which may indicate that the root morphogenesis of halophytes was less affected by soil salt at this stage. However, at the flower bud and fruit stages, TSS was the main limiting factor for root growth (**Figure 7A**), and Na uptake by roots also displayed the curvilinear changes (**Figure 7B**). When soil TSS concentration was approximately 5.00g kg^-1^, Na^+^ uptake reached a maximum of 111.31 mg and 98.71 mg at the flower bud and fruit stages, respectively (**Figure 7B**). Although they were significantly higher than that at the seedling stage when soil TSS content was lower than approximately 15.00 g kg^-1^, Na^+^ uptake at the seedling stage showed an upward trend accompanied by the increase of TSS content, and under the highest salt treatment, it was higher than that at the other two stages, which may be due to the fact that high salt stress severely inhibited root growth and root biomass did not increase with the process of growth and development.

### 3.5 The relationship between soil available N and the biomass of *S. salsa*

**Figure 8:**
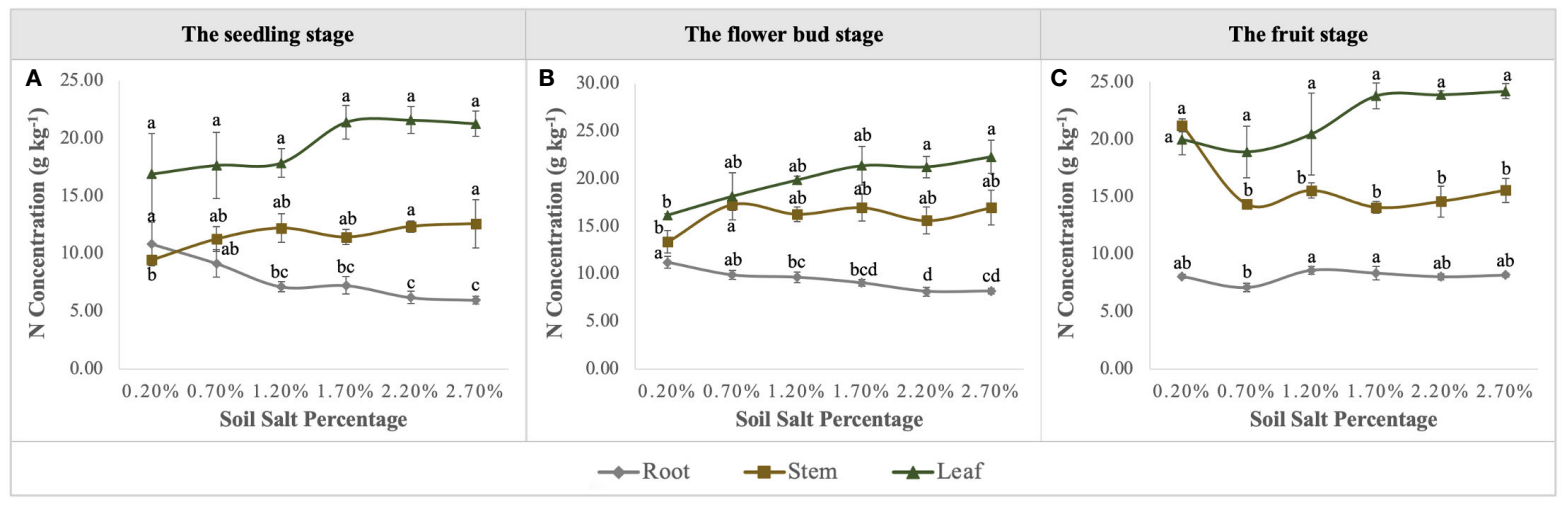
Variations in N concentration in roots, stems, and leaves under six levels of NaCl addition at the seedling **(A)**, flower bud **(B)** and fruit **(C)** stages. Each treatment had five biological replicates and the assays were repeated five times. Vertical error bars indicate ± SD (N = 5). Different lowercase letters indicate statistically significant differences (P < 0.01).

**Figure 9:**
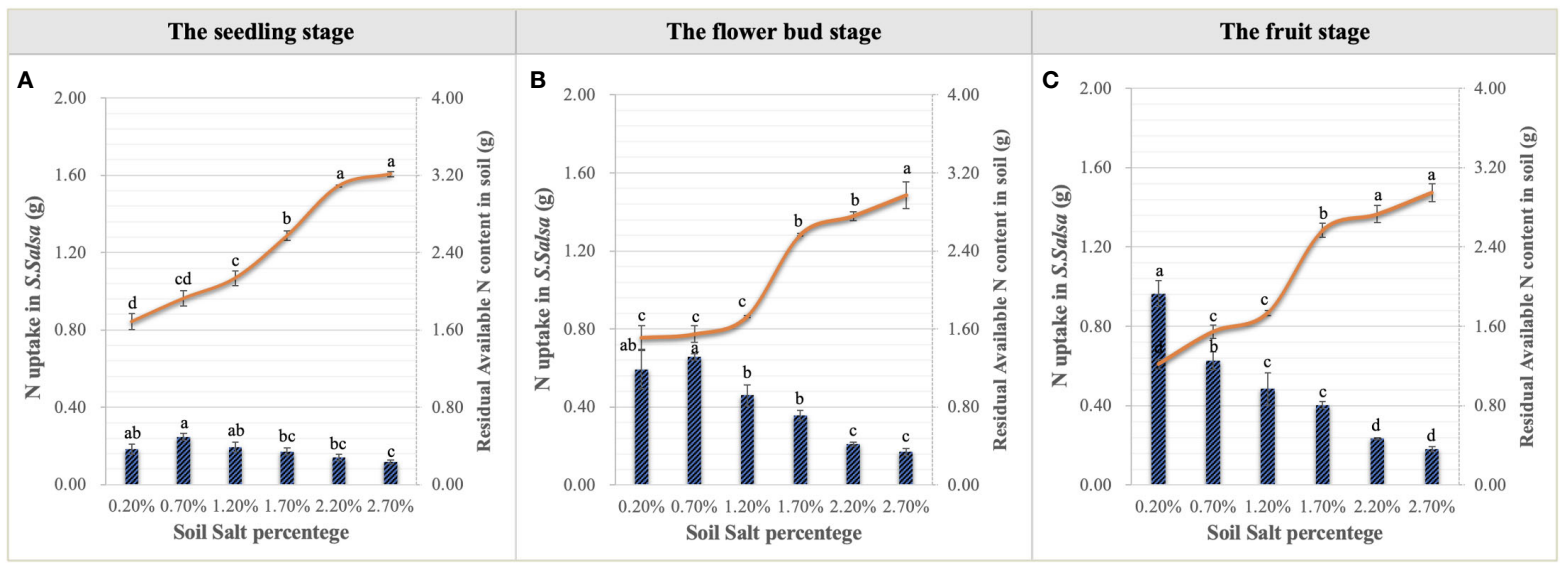
Variations in N uptake in *S. salsa* and soil residual available N content under six levels of NaCl addition at the seedling **(A)**, flower bud **(B)** and fruit **(C)** stages. Each treatment had five biological replicates and the assays were repeated five times. Vertical error bars indicate ± SD (N = 5). Different lowercase letters indicate statistically significant differences (P < 0.01).

Urea applied into the saline-alkali soil would be rapidly transformed into NO_3_^–^N to supply the growth of *S. salsa. S. salsa* grown on the low and medium salt concentration soils (0.20%, 0.70%, and 1.20%) had significant NO_3_^–^N consumption, especially on the topsoil (**Figure 4**). Additionally, during the whole growth period, N concentration of *S. salsa* was basically leaf > stem > root, and it increased with the increase in salt concentration in stems and leaves (**Figures 8A–C**). Therefore, combined with the consumption of soil N, it was inferred that the biomass formation with a low salt concentration mainly depended on N uptake. On the contrary, the high salt concentration restricted N uptake and utilization (**Figures 9A–C**). At the same time, we found that the differences in the N uptake curve at the fruit stage, compared with the other two periods, might be related to the fact that the increment in N concentration in the stem under the control treatment accelerated the growth of the stem and thus promoted biomass accumulation (**Figure 8C**). Additionally, the correlation analysis showed that biomass at the three growth stages was significantly positively correlated with N in the plant, yet negatively correlated with soil TSS and residual available N, i.e., the biomass accumulation curvilinear responses of *S. salsa* to soil salt were attributed to soil N absorption restriction dominated by salt stress (**Table 2**).

**Table 2:** Correlation analysis between biomass and plant N, soil residual available N, and TSS.

	Plant N	Soil residual available N	Total soluble salt
**Biomass at the seedling stage**	0.976**	-0.914*	-0.890*
**Biomass at the flower bud stage**	0.981**	-0.959**	-0.985**
**Biomass at the fruit stage**	0.985**	-0.965**	-0.991**

Each stage had 30 biomass values (mean), plant N (mean), soil residual available N (the weighted mean), and TSS (the weighted mean). Asterisks indicate the significant correlation between biomass and its influencing factors, calculated using a Pearson test (P<0.05*, P<0.01**).

### 3.6 The amount of salt removal from the aboveground part of *S. salsa*

**Figure 10:**
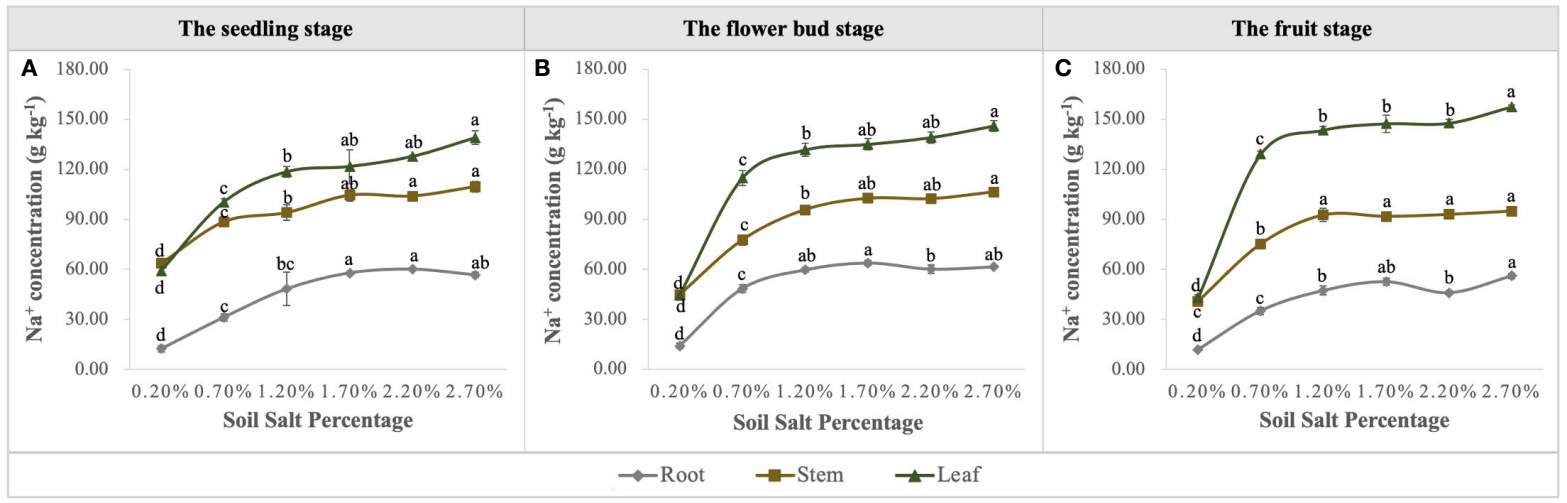
Variations in Na concentration in roots, stems, and leaves under six levels of NaCl addition at the seedling **(A)**, flower bud **(B)** and fruit **(C)** stages. Each treatment had five biological replicates and the assays were repeated five times. Vertical error bars indicate ± SD (N = 5). Different lowercase letters indicate statistically significant differences (P < 0.01).

**Figure 11:**
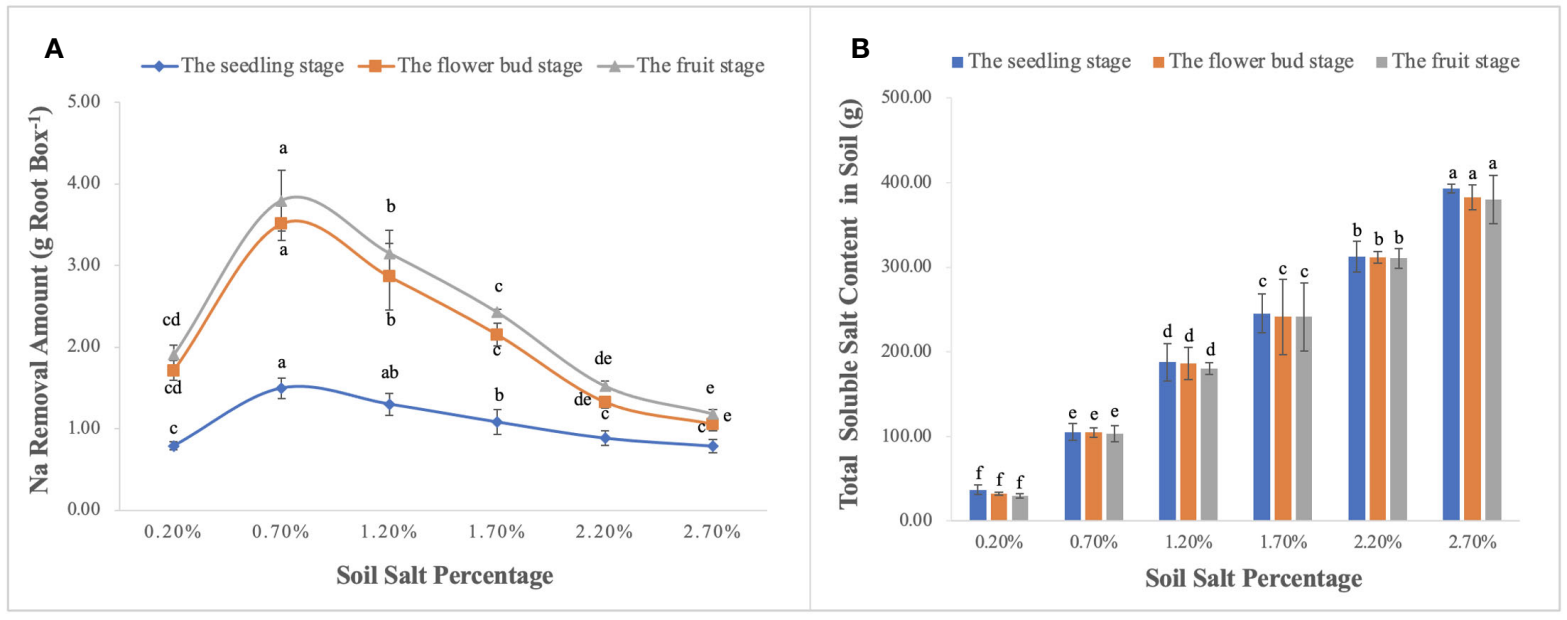
Na removal by the aboveground part of *S. salsa* **(A)** and soil residual TSS content **(B)** during the growth period. Each treatment had five biological replicates and the assays were repeated five times. TSS indicates the weighted mean. Vertical error bars indicate ± SD (N = 5). Different lowercase letters indicate statistically significant differences (P < 0.01).

Na concentration in the vegetative organs was promoted significantly with the increase of soil salt content, and it was ordered as follows: leaf > stem> root (**Figure 10**), i.e., leaves were the main salt-accumulating organs, followed by stems (**Figures 10A–C**). We calculated the biological desalinization by multiplying the Na concentration of leaves and stems by their biomass, and obtained the amount of desalinization of *S. salsa* at the different growth stages, which was in the order of fruit stage > flower bud stage > seedling stage. Moreover, when soil salt content was 0.70%, the amount of Na removal reached 3.80 g per root box, accounting for approximately 4.22% of NaCl addition (**Figure 11A**). Despite the desalting ability of *S. salsa*, the salt removal effect remained minimal as its growth progressed in the pot experiment (**Figure 11B**).

## 4 Discussion

### 4.1 The law of biomass allocation in *S. salsa*

The law of biomass allocation is associated with the growth and metabolism of plants and determined by the function of plant organs (Hecht et al., 2019). For *S. salsa*, leaves are the main salt-storage organs for its growth and survival in a saline habitat (Wang et al., 2023b). In terms of its biomass distribution pattern, more information about the environmental pressures is needed, because plants show remarkable morphological plasticity in response to environmental pressures such as soil nutrients and salinity (Sultan, 2000; Hermans et al., 2006). In this study, the biomass accumulation of *S. salsa* at different stages shows the corresponding curvilinear responses to the increase in soil soluble salt (**Figure 2**). At the seedling stage, a soil salt level of 0.70% creates the optimal soil environment for its growth, with the highest biomass (**Figure 2A**). The result is consistent with a previous study, indicating that 0.50% soil NaCl addition could be the most suitable salt level for *S. salsa* growth (Wang et al., 2021b). At the flower bud stage and fruit stage, a soil salt level of 0.20% (control treatment) harvests the highest biomass and the increment is bigger than those of the other treatments at the later growth stage (**Figures 2A, B**), i.e., salt is not an essential requirement for *S. salsa* in a nutrient-rich environment. However, this finding has only been confirmed in a few euhalophytes, such as *Inula crithmoide*, *Plantago crassifolia*, and *Suaeda glauca*, which grew better under salt stress due to the higher competition for water and nutrients than is the case with non-halophytes (Grigore et al., 2012; Lu et al., 2022). In fact, this viewpoint is also verified in our study on the N uptake and utilization of *S. salsa* (**Figure 7C**) because the aboveground N concentration of *S. salsa* is significantly higher under the 0.20% salt treatment than under other treatments, which explains why the biomass of *S. salsa* obviously decreases with the increased salt content throughout the growth and development period (**Figure 2**). Furthermore, it is necessary for plants to balance aboveground and belowground biomass allocation to maintain their normal growth and improve their adaptability to the environment (Shipley and Meziane, 2002; Mensah et al., 2016). *S. salsa* balances the biomass allocation through increasing the leaf mass ratio, decreasing the stem mass ratio, and stabilizing the root mass ratio to acclimate to the varied soil soluble salts (**Figure 3**), and this allocation pattern of each organ was maintained throughout the three growth stages (**Figures 3A, D, G**). In addition, the aboveground biomass of *S. salsa* is much greater than the belowground biomass, which is consistent with a previous study (Wang et al., 2022) and conducive to the transfer of soil soluble salt to the plant. *S. salsa* under a salt level of 2.20% had a similar biomass accumulation and allocation of *S. salsa* under 2.20% salt level was similar to that under the 2.70% salt level. Meanwhile, 2.70% salt led to their withering and death at the fruit stage (**Figure 1B**). Therefore, 2.50% NaCl could be regarded as the critical concentration at which *S. salsa* can tolerate a salinized habitat.

### 4.2 The response of root morphology to soil soluble salt

Plant morphological traits can be considered as potential covariates for comprehending biomass allocation (Mensah et al., 2016; Yin et al., 2019; Wang et al., 2022). In saline habitats, the root system of *S. salsa* is not only responsible for water, salt, and nutrient acquisition, but is probably subjected to the adverse effects of salt stress, such as osmotic stress and ion toxicity (Kang and Zhang, 2004; Ma et al., 2012). Thus, RL and its distribution, as well as RAD, can be deemed to be important morphological indicators that reflect root adaptive responses to soil salinity (Wang et al., 2021b). At the seedling stage, root formation is the main behavior of the root system, RL and RAD are significantly improved under the low salt and medium salt levels, while they have no significant differences under the high salt treatments (**Figures 5A**, **6**), which may be related to salt supplementary after the seedlings growing for a period of time, when the root system was initially established. After the emergence of *S. salsa*, NaCl application at intervals might weaken ion toxicity on the roots. Another explanation may be that the root systems of *S. salsa* have a potential salt-resist mechanism, i.e., they can adjust root architecture and structure, such as root elongation (Wang et al., 2021b; Lupo et al., 2022; Fan et al., 2023; Keriman et al., 2024), or attract salt-tolerant microbial colonization via root exudates (Xiong et al., 2020; Niraj et al., 2023) to ensure its normal growth under salt stress. With the growth and development of *S. salsa*, the flower bud stage becomes the most active period of root growth. In this period, RLs in different soil layers and the RADs of the taproots increased to some extent. However, total RL under a salt level of 2.50% decreased, indicating that extreme salt levels significantly inhibit root growth (**Figures 5B**, **6**). At the fruit stage, salt significantly accelerated the damage and death of roots, while salt concentrations of 1.20% and 1.70% significantly increased the RADs of taproots (**Figures 5C**, **6**). High salt concentrations may have caused the thinner lateral roots originating from taproots to fall off, while the low salt levels (0.20% and 0.70%) made lateral roots share the mean of taproot RAD, thereby indirectly reducing the RAD. Consequently, RADs at the flower bud and fruit stages could not reflect the real morphological characteristics of taproots on topsoil. However, the changes of RAD at the seedling stage can provide some valuable references for the real root phenotypic responses to soil soluble salt levels.

**Figure 12:**
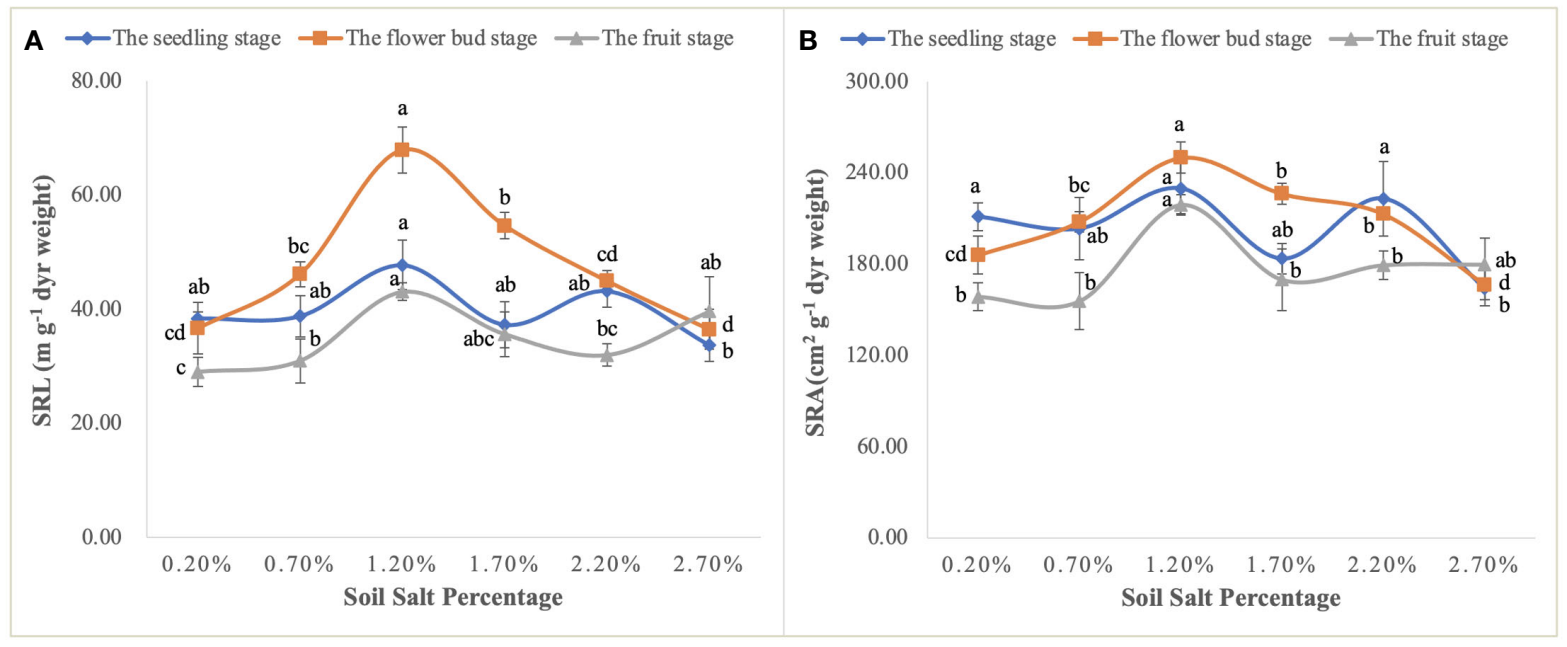
Variations in SRL **(A)** and SRA **(B)** at the seedling, flower bud, and fruit stages. Each treatment had five biological replicates and the assays were repeated five times. Vertical error bars indicate ± SD (N = 5). Different lowercase letters indicate statistically significant differences (P < 0.01).

Moreover, the curvilinear regression of RL to soil TSS further confirms the effect of salinity on root architecture mentioned above (**Figure 7A**), and the curvilinear responses of Na accumulation in root to soil TSS shows that the root could absorb more salt under the light salt (0.70%) and moderate salt (1.20%) levels at the flower bud stage (**Figure 6B**), which can be attributed to the higher sodium absorption capacity of the root system during this period (**Figure 9**). In summary, a series of soil soluble salts, at low, moderate, heavy, and excessive salt concentrations, caused the curvilinear responses of the root morphology, which was reported in a previous study (Wang et al., 2021b). Furthermore, we found that the salt concentration that leads to the optimal RL architecture is not exactly consistent with the salt concentration that produces the maximum biomass. Specific root length (SRL) and specific root area (SRA) are calculated by dividing total root length and total root area by root mass, respectively. They are important indices of plant root morphology and growth that can be used to quantify the development status of the root system, the capacity of nutrient and water acquisition, and ecological adaptation (Ahmadi et al., 2018). In this study, SRL and SRA were highest under a salt level of 1.20%, indicating that this salt concentration can stimulate the root system to produce more fine and hairy roots to access more soil water and nutrients (**Figure 12**).

### 4.3 The relationship between aboveground biomass and phytodesalination dominated by salt and nitrogen

Under an adequate N supply, the biomass response of euhalophytes to different salt gradients can determine its ecological value for saline-alkali land improvement. Previous studies have shown that the biomass response of *S. salsa* to soil salt gradients (ranges from non-saline soil to heavy saline soil) is of a curvilinear type (Flowers and Colmer, 2008; Ma et al., 2019), and biomass is at its maximum when soil NaCl content accounts for approximately 0.50% of the soil weight (Chapman, 1942; Wang et al., 2021b). However, this study clearly shows that *S. salsa* grows best in non-saline soil (a soil salt level of 0.20%) when soil N and phosphorus are sufficient (**Figures 1B**; **2B, C**). At the fruit stage in particular, a rapid increase in biomass may be attributed to more complete root architecture, yet the presence of salt accelerates root decay and reduces root length during this period (**Figures 5**, **6A**). Concurrently, this result supports previous studies with regard to whether the growth and development of euhalophytes depend on salt, suggesting that some euhalophytes grow better in nutrient-rich and salt-absent soil (Grigore et al., 2012; Lu et al., 2022). In addition, the correlation between biomass and plant N content, soil residual N, and TSS over the whole growth period reveals that an adequate N supply can significantly increase biomass, whereas TSS significantly decreases biomass (**Table 2**).

The other essential parameter for evaluating phytodesalination was Na concentration in the aboveground organs of *S. salsa*. In this study, Na^+^ concentration increased with the progressive salt gradients, and the order of Na concentration in each organ was leaf > stem > root, which is consistent with previous studies (Wang et al., 2021b). The amount of salt accumulation obtained by multiplying the biomass of aboveground organs by its corresponding Na^+^ concentration is shown in **Figure 11A**, and the quantity of phytodesalination responds to salt gradients in the form of curves; the optimum is obtained in the light-saline soil, followed by moderate-saline soil. Additionally, phytodesalination is associated with harvest time, and harvest after seed maturity and before leaves fall off may be the best choice (Wang et al., 2023a, 2023c). Although *S. salsa* has greater ecological value in phytodesalination than other euhalophytes, the desalting amount accounted for only a small fraction of soil TSS in the pot trails (**Figure 10B**), let alone in the field. Therefore, another consideration for soil salt removal in saline-alkali land should be focused on exploring the ecological function of root systems with regard to soil water-salt transport under local irrigation models (Yang et al., 2019; Wang et al., 2021a) to reduce salt in the surface soil for the normal growth of crops.

## 5 Conclusion

Throughout the entire growth and development of *S. salsa*, its responses of biomass, root morphological adjustments, and phytodesalination capacity to soil soluble salt undoubtedly present the curvilinear characteristics, and these responses are predominantly caused by the differential absorption and utilization of salt and nutrients (particularly N). Thus, we summarized the law of biomass allocation for *S. salsa*. With the increase in soil soluble salt, the leaf mass ratio increased significantly, the stem mass ratio decreased significantly, and the root mass ratio increased slightly. For individuals subjected to the same salt level, leaf mass ratio > stem mass ratio > root mass ratio (except the control treatment at the flower bud and fruit stages). Moreover, based on the biomass responses, the relationship between biomass and soil environment factors can be elaborated as follows: soil TSS significantly inhibits the growth of *S. salsa*, whereas N significantly promotes its growth. Additionally, salt is not strictly required for *S. salsa* when there is an adequate N supply, but this conclusion need to be proved further.

The biomass and ability of *S. salsa* to acquire salt jointly determine its phytodesalination capacity on saline-alkali land. Therein, root morphological adjustment is a critical factor that affects its access to salt and nutrients. The responses of RL and RAD to soil TSS show the curvilinear types, and the highest point of the curves correspond to salt concentration in the light-saline soil. In addition, at the fruit stage of *S. salsa*, soil soluble salt can accelerate root aging and death, thereby restricting nutrient acquisition to increase biomass. Consequently, based on the responses of biomass accumulation and root morphology, we draw the conclusion that aboveground phytodesalination is the best in light saline soil, and the specific root length is largest in moderate saline soil, which cannot be ignored in future studies on the effects of root architecture on soil water and salt transport.

## Data availability statement

The raw data supporting the conclusions of this article will be made available by the authors, without undue reservation.

## Author contributions

YW: Conceptualization, Investigation, Methodology, Visualization, Writing – original draft. TG: Conceptualization, Supervision, Writing – review & editing. CT: Funding acquisition, Methodology, Project administration, Validation, Writing – review & editing. ZZ: Conceptualization, Investigation, Writing – review & editing. KZ: Investigation, Supervision, Writing – review & editing. WM: Conceptualization, Funding acquisition, Methodology, Project administration, Validation, Writing – review & editing.
